# Factors associated with female genital mutilation among women of reproductive age and girls aged 0–14 in Chad: a mixed-effects multilevel analysis of the 2014–2015 Chad demographic and health survey data

**DOI:** 10.1186/s12889-021-10293-y

**Published:** 2021-02-04

**Authors:** Bright Opoku Ahinkorah

**Affiliations:** School of Public Health, Faculty of Health, University of Technology Sydney, Sydney, NSW Australia

**Keywords:** Female genital mutilation, Girls, Chad, DHS, Global Health

## Abstract

**Background:**

Chad is one of the African countries with high prevalence of female genital mutilation (FGM). The aim of this study was to examine the factors associated with FGM among women aged 15–49 and girls aged 0–14 in Chad.

**Methods:**

Data for the study were obtained from the 2014–2015 Chad Demographic and Health Survey. FGM among women aged 15–49 and girls aged 0–14 were the outcome variables. The prevalence of FGM among women and girls were presented using percentages while a mixed-effects multilevel multivariable logistic regression analysis was carried out to assess the factors associated with FGM. The results were presented using adjusted odds ratio with associated 95% confidence intervals.

**Results:**

The results indicate that more than half (50.2%) of the women and 12.9% of girls in Chad had been circumcised. Among women aged 15–49, level of education, employment status, ethnicity, religion, wealth quintile and community literacy level were significant predictors of FGM. Age, partner’s level of education, marital status, employment status, ethnicity, religion and mother’s FGM status were associated with FGM among girls aged 0–14.

**Conclusion:**

This study has identified several individual and contextual factors as predictors of FGM among women and girls in Chad. The findings imply the need to adopt strategies aimed at addressing these factors in order to help eliminate the practice of FGM. Government and non-governmental organisations in Chad need to implement policies that enhance media advocacy and community dialogue to help deal with FGM in the country.

## Background

Female genital mutilation (FGM) involves the partial or total removal of external female genitalia or other injury to the female genital organs for non-medical reasons [1]. Predominantly, it is conducted in Africa and the Middle East [2] and on young females between infancy and adolescence [1]. According to UNICEF [3], over 200 million girls and women alive today have undergone some form of FGM and 30 million girls are at risk of experiencing FGM in the next decade. FGM is associated with several complications which include severe pain (usually in the absence of anaesthetic agents), acute urinary retention, vaginal lacerations at coitus and haemorrhage [1, 4, 5]. In the long term, FGM may result in poor quality of life, death or both [6].

In Africa, FGM is practiced in 29 countries, with variations in terms of the proportion of girls and women who have undergone FGM in each country [7]. Two indicators have been used to measure the prevalence of FGM [7]. The first indicator reports on the prevalence of the practice as the percentage of girls and women of reproductive age (15–49) who have experienced any form of FGM [7]. For instance, in countries such as Mali, Guinea, Sierra Leone, Sudan, Egypt and Somalia, the percentage of women aged 15–49 who have undergone FGM is above 80%. In Ethiopia, Mauritania, Burkina Faso and Liberia, the proportion of women aged 15–49 who have undergone FGM is between 51 and 80% whiles in chad, 44% of women have undergone FGM [7].

The second indicator reports on FGM among daughters of women of reproductive age (15–49). This indicator is used to measure the prevalence of FGM among girls aged 0 to 14 [7]. Using this indicator, the prevalence of FGM in some sub-Saharan African countries such as Burkina Faso was 13% in 2010, 23.4% in Ethiopia in 2011, 56% in Gambia in 2010, 45% in Guinea in 2016, 3% in Kenya in 2014, 75% in Mali in 2014, 17% in Nigeria in 2013 and 15% in Senegal in 2015 [8]. A recent systematic analysis of pooled data from 29 countries spread across Africa and two countries in Western Asia found the practice of FGM among girls 0–14 to be high in a number of African countries including Chad. In that study, Chad was the country with the highest prevalence of FGM among girls 0–14 (13.9%) in Central Africa [9]. According to the 2014–15 Chad Demographic and Health Survey, 10% of girls aged 0–14 have been circumcised [10]. This indicates that literature on the practice of FGM among girls aged 0 to 14 is essential.

Despite the relatively high prevalence of FGM among women and girls in Chad, no study has examined the predictors of FGM among women aged 15–49 and girls aged 0–14 in the country. Hence, the aim of this study was to examine the factors associated with FGM among women aged 15–49 and girls aged 0–14 in Chad. Findings from this study will help develop strategic interventions and approaches in eliminating FGM in the country.

## Methods

### Data

Data for this study was obtained from the 2014–2015 Chad Demographic and Health Survey (DHS), which is part of the MEASURE DHS Program and aims at obtaining information on a number of population and health issues including FGM. Taking households as sampling units, the survey employed a multi-stage, stratified sampling design to select all eligible women for interviews [10]. For this study, the women’s file which contains data of women of reproductive age (15–49) was considered. A sample of 6334 married and cohabiting women who had daughters and had complete cases on all the variables of interest in this study was considered.

### Study variables

#### Outcome variable

Two outcome variables were considered in this study. The first outcome variable was “FGM among women” and the second outcome variable was ‘FGM among girls aged 0-14’. To derive FGM among women, respondents were asked if their genital area was “nicked with nothing removed,” “something removed,” or “sewn shut”. The responses were coded as ‘Yes’ and ‘No’. FGM among girls aged 0–14 was obtained by asking women who had daughters the question “how many of your daughter(s) had their genital area “nicked with nothing removed;” “something removed,” or “sewn shut”? The responses ranged from ‘no daughter’ to ‘1, 2, 3, 4, 5, 6, 7 daughters’. A binary outcome was then created from these responses with those who said none of their daughters went through FGM coded as ‘No’ and those who had at least one daughter circumcised coded ‘Yes’. The coding of these variables were in line with previous studies [11–13].

#### Explanatory variables

Fifteen explanatory variables, made up of twelve individual level factors and three contextual factors were used in this study. The individual level factors were age, mother’s education, partner’s education, marital status, employment status, ethnicity, religion, exposure to media (newspaper/magazine, radio and television), mother’s circumcision status and wealth quintile. Age was categorized into “15-24, 25-34 and 35 years and above”. Mother’s and partner’s education were categorized as “no education, primary and secondary or higher education”. Employment status was grouped into “employed or unemployed” and marital status was categorized into “married and ohabiting”. Christianity, Islam and others (animist, other religion and no religion) were used to define religion and ethnicity was categorised into “Boulala/medego/kouka”, “Ouaadai/maba/massalit/mimi”, “Gorane”, “Sara,” “Arab”, “Massa/mousseye/mousgoume”, “Kenemu-Borno”, and “other” (other Beninois and other nationalities). Exposure to media (newspaper/magazine, radio and television) were each categorized into “not at all, less than once a week and at least once a week.” Mother’s circumcision status was categorized as “yes or no” and wealth quintile as “poorest, poorer, middle, richer, richest”. The contextual factors were place of residence (urban or rural), community literacy level- proportion of women who can read and write (low, medium, high) and community socio-economic status- proportion of women in the richest household quintile (low, medium, high). The selection of these variables was influenced by their associations with FGM in previous studies [11, 12, 14–16].

### Statistical analyses

The analyses began with the calculation of the prevalence of FGM among women and girls aged 0–14, together with a distribution of FGM among women and girls aged 0–14 across the individual and contextual level factors using percentages. Chi-square test of independence was used to assess the statistical significance of the association between each of the explanatory variables and FGM among women and girls aged 0–14 at a *p*-value of 0.05 (see Table 1). This was followed by a two-level multilevel multivariable logistic regression analysis that examined the individual and contextual factors associated with FGM among women and girls 0–14 years. Variables that showed significant associations with FGM at the chi-square level were used in the modelling for the multilevel multivariable logistic regression analysis. The use of the two-level modelling implies that women were nested within clusters (primary sampling units). The clusters were used to address the unexplained variability at the contextual level for the random effects analysis [17].

**Table 1:** Socio-demographic characteristics of women and female genital mutilation among women aged 15–49 and girls aged 0–14 in Chad (Weighted, *N* = 6334)

Variables	Frequency (n)	Percentage (%)	FGM in women aged 15–49	χ2 (p-value)	FGM in girls aged 0–14	χ2 (p-value)
**Individual level factors**						
**Age**				0.40 (0.818)		392.2 (< 0.001)
15–24	1884	29.7	48.6		1.2	
25–34	2530	40.0	51.0		14.5	
35 years and above	1920	30.3	50.8		22.3	
**Mother’s education**				150.7 (< 0.001)		172.6 (< 0.001)
No education	4290	67.7	56.8		16.9	
Primary	1382	21.8	39.5		5.3	
Secondary/Higher	662	10.5	30.2		3.0	
**Partner’s education**				171.5(< 0.001)		214.6 (< 0.001)
No education	3768	59.5	58.1		18.5	
Primary	1179	18.6	42.3		6.9	
Secondary/Higher	1387	21.9	36.4		2.7	
**Marital status**				10.1 (0.001)		33.1 (< 0.001)
Married	5757	90.9	51.4		13.9	
Cohabiting	577	9.1	38.6		3.3	
**Employment status**				29.0 (< 0.001)		15.5 (< 0.001)
Not working	2995	47.3	48.8		12.6	
Working	3339	52.7	51.5		13.2	
**Ethnicity**				7.6 (< 0.001)		497.04 (< 0.001)
Sara	2065	32.6	44.8		4.6	
Gorane	256	4.0	11.5		3.9	
Arab	833	13.2	89.8		24.9	
Ouaadai/maba/massalit/mimi	647	10.2	82.0		30.2	
Kenemu-Borno	446	7.1	10.2		3.1	
Boulala/medego/kouka	282	4.5	61.3		17.1	
Other	1804	28.5	40.5		13.6	
**Religion**				256.1 (< 0.001)		233.3 (< 0.001)
Christianity	2628	41.5	35.1		3.7	
Islam	3583	56.6	62.2		20.0	
Other	123	2.0	30.0		4.1	
**Frequency of reading newspaper/magazine**				53.6 (< 0.001)		43.9 (< 0.001)
Not at all	5913	93.3	51.7		13.6	
Less than once a week/at least once a week	421	6.7	29.6		3.0	
**Frequency of listening to radio**				47.6 (< 0.001)		44.3 (< 0.001)
Not at all	4689	74.0	53.2		14.5	
Less than once a week/at least once a week	1645	26.0	41.8		8.5	
**Frequency of watching television**				17.5 (< 0.001)		26.7 (< 0.001)
Not at all	5582	88.1	51.3		13.6	
Less than once a week/at least once a week	752	11.9	42.5		7.7	
**Respondent circumcised**				N/A		974.5 (< 0.001)
No	3153	49.8	N/A		0.4	
Yes	3181	50.2	N/A		25.3	
**Wealth quintile**				102.4 (< 0.001)		32.9 (< 0.001)
Poorest	1590	21.0	57.1		14.1	
Poorer	1328	20.9	53.2		12.7	
Middle	1320	20.2	47.9		13.9	
Richer	1280	18.4	46.5		14.4	
Richest	1238	19.5	45.5		9.3	
**Contextual factors**						
**Place of residence**				2.1 (0.147)		17.3 (< 0.001)
Urban	1390	22.0	49.4		10.3	
Rural	4944	78.0	50.6		13.6	
**Community literacy level**				170.2 (< 0.001)		180.7 (< 0.001)
Low	2271	35.9	60.6		20.3	
Medium	2057	32.5	45.0		11.0	
High	2007	31.8	40.7		6.4	
**Community socio-economic status**				103.2 (< 0.001)		58.4 (< 0.001)
Low	4025	63.5	53.4		14.6	
Medium	575	9.1	40.1		8.9	
High	1734	27.4	44.1		10.4	

Source: 2014–2015 Chad Demographic and Health Survey

NB: N/A indicates that prevalence for FGM and chi-square test results were not applicable for that variable

In the multilevel multivariable modelling, four models, comprising of the empty model (model 0), model 1 model 2 and model 3 were fitted. Model 0 was fitted to show the variance in FGM in women and girls which could be attributed to the clusters without the explanatory variables. Model 1 showed the interaction between the individual-level factors and FGM among women and girls. Model 2 had the contextual level factors and FGM among women and girls while Model 3 contained the individual and contextual level factors and FGM among women and girls. Stata version 14.2 for windows was used in analyzing the data. The Stata command “melogit” was used in fitting these models. The models were compared using the log-likelihood ratio (LLR) and Akaike’s Information Criterion (AIC) tests. The results of the fixed effect analysis were presented using adjusted odds ratios (aOR) and their 95% confidence intervals (CIs) (see Table 2 and 3). To check for high correlation among the explanatory variables, a test for multicollinearity was carried out using the variance inflation factor (VIF) and the results showed no evidence of high collinearity. Sample weight (v005/1,000,000) was used to correct for over and under-sampling while the SVY command was used to address the complex survey design and generalizability of the findings.

**Table 2:** Multivariable multilevel logistic regression models on individual and contextual factors associated with circumcision among women aged 15–49 in Chad

Variables	Model 0	Model 1	Model 2	Model 3
		aOR [95% CI]	aOR [95% CI]	aOR [95% CI]
**Individual level factors**				
**Mother’s education**				
No education		Ref		Ref
Primary		0.79[0.60–1.04]		0.81[0.61–1.07]
Secondary/Higher		0.44^***^[0.28–0.68]		0.45^***^[0.29–0.71]
**Partner’s education**				
No education		Ref		Ref
Primary		0.95[0.71–1.29]		0.99[0.73–1.34]
Secondary/Higher		0.82[0.60–1.13]		0.85[0.62–1.16]
**Marital status**				
Married		Ref		Ref
Cohabiting		1.00[0.68–1.48]		1.00[0.68–1.48]
**Employment status**				
Not working		0.80^*^[0.64–0.99]		0.79^*^[0.64–0.99]
Working		Ref		Ref
**Ethnicity**				
Sara		Ref		Ref
Gorane		0.005^***^[0.002–0.011]		0.005^***^[0.002–0.010]
Arab		1.13[0.60–2.12]		1.08[0.58–2.03]
Ouaadai/maba/massalit/mimi		0.81[0.40–1.62]		0.74[0.37–1.49]
Kenemu-Borno		0.012^***^[0.001–0.025]		0.011^***^[0.005–0.023]
Boulala/medego/kouka		0.17^***^[0.08–0.36]		0.17^***^[0.08–0.36]
Other		0.13^***^[0.06–0.17]		0.12^***^[0.07–0.20]
**Religion**				
Christianity		0.10^***^[0.06–0.17]		0.12^***^[0.07–0.19]
Islam		Ref		Ref
Other		0.09^***^[0.03–0.21]		0.10^***^[0.04–0.24]
**Frequency of reading newspaper/magazine**				
Not at all		Ref		Ref
Less than once a week/at least once a week		0.98[0.61–1.58]		0.95[0.59–1.54]
**Frequency of listening to radio**				
Not at all		Ref		Ref
Less than once a week/at least once a week		0.95[0.72–1.25]		0.98[0.74–1.29]
**Frequency of watching television**				
Not at all		Ref		Ref
Less than once a week/at least once a week		1.28[0.88–1.84]		1.34[0.93–1.94]
**Wealth quintile**				
Poorest		Ref		Ref
Poorer		0.75[0.56–1.02]		0.75[0.56–1.02]
Middle		0.73[0.53–1.01]		0.73[0.53–1.01]
Richer		0.67^*^[0.47–0.94]		0.68^*^[0.48–0.96]
Richest		0.65[0.40–1.05]		0.88[0.51–1.52]
**Contextual factors**				
**Community literacy level**				
Low			Ref	Ref
Medium			0.25^***^[0.12–0.54]	0.42^**^[0.22–0.80]
High			0.09^***^[0.04–0.19]	0.27^**^[0.13–0.54]
**Community socio-economic status**				
Low			Ref	Ref
Medium			0.83[0.21–3.33]	0.86[0.55–1.34]
High			1.26[0.60–2.65]	0.97[0.48–1.94]
**Random effect result**				
PSU variance (95% CI)	19.6 (15.0–25.5)	8.1 (6.5–10.1)	18.4 (14.2–23.7)	7.99 (6.41–9.95)
ICC	85.6%	71.2%	84.8%	70.8%
LR Test	χ^2^ = 3915.41, p < 0.001	χ^2^ = 1815.97, p < 0.001	χ^2^ = 3742.84, *p* < 0.001	χ^2^ = 1801.56, p < 0.001
Wald χ^2^	Reference	491.58***	46.41**	509.50***
Model fitness				
Log-likelihood	− 2432.46	− 2149.31	− 2420.83	− 2141.16
AIC	4868.93	4344.62	4853.66	4336.32
N	6334	6334	6334	6334

Source: 2014–2015 Chad Demographic and Health Survey

Exponentiated coefficients; 95% confidence intervals in brackets

^*^ *p* < 0.05, ^**^ *p* < 0.01, ^***^ *p* < 0.001

*N* Sample size, *Ref* Reference category, *PSU* Primary Sampling Unit; *ICC* Intra-Class Correlation, *LR Test* Likelihood ratio Test, *AIC* Akaike’s Information Criterion

## Results

### Prevalence of female genital mutilation among women and girls 0–14 years in Chad

**Fig. 1:**
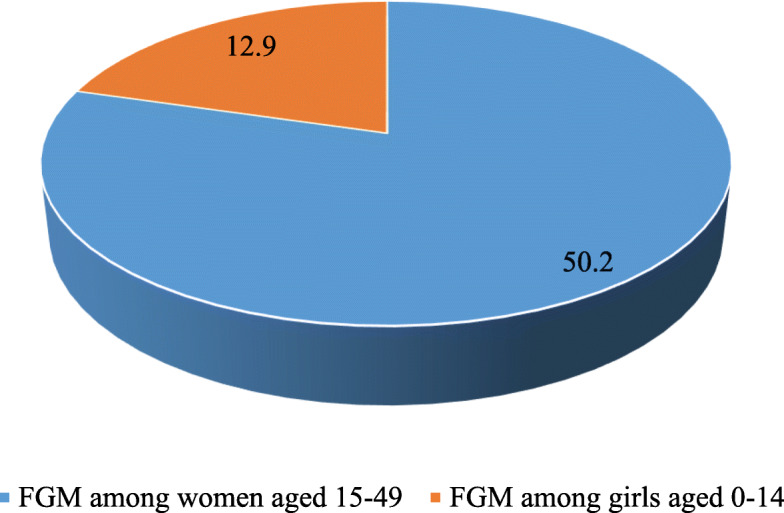
Prevalence of FGM among women aged 15–49 and girls aged 0–14 in Chad

Figure 1 shows the prevalence of FGM among women and girls aged 0–14 in Chad. The results indicate that more than half (50.2%) of the women and 12.9% of girls aged 0–14 in Chad had been circumcised.

### Distribution of FGM among women and girls aged 0–14 per the socio-demographic characteristics of women

Out of the 6334 women involved in this study, 40% were aged 25–34, 67.7% had no formal education, 59.5% had partners with no formal education, 90.9% were married, 52.7% were working, 32.6% belonged to the Sara ethnic group and 56.6% were Muslims. Again, 93.3% of the women never read newspaper/magazine, 74.0% never listened to radio, 88.1% never watched television, 50.2% were circumcised, 21% belonged to the poorest wealth quintile, 78% were in rural areas, 35.9% were in communities with low literacy level and 63.5% were in communities with low socio-economic status. All the explanatory variables had significant relationship with circumcision of girls at 95% CI. However, age and place of residence had no significant associations with FGM among women (Table 1).

### Predictors of female genital mutilation among women aged 15–49 in Chad

Results of the multivariable multilevel logistic regression analysis, as shown on Model 3, indicate that the likelihood of FGM was lower among women who had secondary/higher education (aOR = 0.45, CI = 0.29–0.71), compared to those who had no formal education. Compared with working women, a lower odd of FGM was found in non-working women (aOR = 0.79, CI = 0.64–0.99). Compared to women of the Sara ethnic group, the odds of FGM was lower among those of “Gorane”, “Kanemu-Borno”, “Boulala/medego/kouka” and other ethnic groups. Compared to Muslims, Christian women were less likely to be circumcised (aOR = 0.12, CI = 0.07–0.19). Women of the richer wealth quintile were less likely to experience FGM compared to the poorest (aOR = 0.68, CI = 0.48–0.96) and the odds of FGM among women was lower in high literacy communities compared to low literacy communities (aOR = 0.27, CI = 0.13–0.54) (Table 2).

### Predictors of female genital mutilation among girls aged 0–14 in Chad

Results of the multivariable multilevel logistic regression analysis, as shown on Model 3, indicate that women aged 25–34 (aOR = 15.29, CI = 10.25–22.79) and those 35 years and above (aOR = 31.72, CI = 21.07–47.77) had higher odds of having circumcised daughters compared to those aged 15–24. The likelihood of FGM among girls was lower among women whose partners had secondary/higher education (aOR = 0.39, CI = 0.25–0.63), compared to those whose partners had no formal education. Compared with working women, a lower odd of FGM among girls was found in non-working women (aOR = 0.77, CI = 0.63–0.95). Women who belonged to Kenemu-Borno ethnic group had the highest odds of having their daughters circumcised compared with those of the Sara ethnic group (aOR = 2.57, CI = 1.27–5.22). Compared to Muslims, Christian women were less likely to get their daughters circumcised (aOR = 0.51, CI = 0.26–0.98). Women who had never experienced FGM, were less likely to have their daughters circumcised (aOR = 0.01, CI = 0.005–0.018), compared to those who had experienced FGM (Table 3).

**Table 3:** Multivariable multilevel logistic regression models on individual and contextual factors associated with circumcision among girls aged 0–14 in Chad

Variables	Model 0	Model 1	Model 2	Model 3
		aOR [95% CI]	aOR [95% CI]	aOR [95% CI]
**Individual level factors**				
**Age**				
15–24		Ref		Ref
25–34		15.30^***^ [10.26–22.80]		15.29^***^ [10.25–22.79]
35 years and above		31.64^***^ (21.02–47.65]		31.72^***^ [21.07–47.77]
**Mother’s education**				
No education		Ref		Ref
Primary		0.94[0.65–1.35]		0.93[0.65–1.35]
Secondary/Higher		0.74[0.32–1.71]		0.76[0.32–1.77]
**Partner’s education**				
No education		Ref		Ref
Primary		0.91[0.64–1.28]		0.92[0.65–1.29]
Secondary/Higher		0.39^***^[0.24–0.62]		0.39^***^[0.25–0.63]
**Marital status**				
Married		Ref		Ref
Cohabiting		0.63[0.36–1.11]		0.63[0.36–1.11]
**Employment status**				
Not working		0.77^*^[0.63–0.94]		0.77^*^[0.63–0.95]
Working		Ref		Ref
**Ethnicity**				
Sara		Ref		Ref
Gorane		1.38[0.49–3.93]		1.41[0.49–4.01]
Arab		1.90[0.96–3.78]		1.96[0.98–3.92]
Ouaadai/maba/massalit/mimi		2.51^**^[1.25–5.06]		2.57^**^[1.27–5.22]
Kenemu-Borno		1.35[0.48–3.74]		1.42[0.50–3.98]
Boulala/medego/kouka		1.67[0.76–3.70]		1.73[0.78–3.84]
Other		1.97^*^[1.03–3.78]		2.05^*^[1.06–3.95]
**Religion**				
Christianity		0.49^*^[0.26–0.92]		0.51^*^[0.26–0.98]
Islam		Ref		Ref
Other		0.35[0.10–1.22]		0.36[0.10–1.23]
**Frequency of reading newspaper/magazine**				
Not at all		Ref		Ref
Less than once a week/at least once a week		0.60[0.23–1.56]		0.62[0.23–1.62]
**Frequency of listening to radio**				
Not at all		Ref		Ref
Less than once a week/at least once a week		1.08[0.80–1.45]		1.10[0.82–1.49]
**Frequency of watching television**				
Not at all		Ref		Ref
Less than once a week/at least once a week		1.01[0.65–1.57]		1.04[0.67–1.62]
**Respondent circumcised**				
No		0.01^***^ [0.005–0.018]		0.01^***^ [0.005–0.018]
Yes		Ref		Ref
**Wealth quintile**				
Poorest		Ref		Ref
Poorer		0.98[0.74–1.29]		0.97[0.73–1.28]
Middle		1.11[0.84–1.48]		1.10[0.83–1.47]
Richer		1.12[0.84–1.52]		1.15[0.85–1.56]
Richest		0.73[0.49–1.08]		0.97[0.56–1.68]
**Contextual factors**				
**Residence**				
Urban			2.17^*^[1.19–3.93]	Ref
Rural			Ref	0.83[0.51–1.33]
**Community literacy level**				
Low			Ref	Ref
Medium			0.37^***^[0.25–0.55]	1.06[0.78–1.45]
High			0.15^***^[0.09–0.26]	0.79[0.50–1.25]
**Community socio-economic status**				
Low			Ref	Ref
Medium			0.50^*^[0.27–0..91]	0.86[0.55–1.34]
High			0.75[0.42–1.35]	0.99[0.62–1.58]
**Random effect result**				
PSU variance (95% CI)	2.78 (2.17–3.56)	0.28 (0.15–0.50)	2.19 (1.70–2.83)	0.27 (0.15–0.50)
ICC	45.8%	7.8%	40.0%	7.6%
LR Test	χ^2^ = 584.12, p < 0.001	χ^2^ = 20.09, *p* < 0.001	χ^2^ = 443.51, p < 0.001	χ^2^ = 19.80, *p* < 0.001
Wald χ^2^	Reference	617.20***	71.91**	617.82***
Model fitness				
Log-likelihood	− 2287.89	− 1539.47	− 2250.53	−1537.37
AIC	4579.79	3130.94	4515.06	3136.74
N	6334	6334	6334	6334

Source: 2014–2015 Chad Demographic and Health Survey

Exponentiated coefficients; 95% confidence intervals in brackets

^*^ *p* < 0.05, ^**^ *p* < 0.01, ^***^ *p* < 0.001

*N* Sample size, *Ref* Reference category, *PSU* Primary Sampling Unit, *ICC* Intra-Class Correlation, *LR Test* Likelihood ratio Test, *AIC* Akaike’s Information Criterion

## Discussion

FGM is predominantly conducted in Africa and the Middle East [2] and on young females between infancy and adolescence [1]. This study sought to examine the predictors of FGM among women aged 15–49 and girls aged 0–14 in Chad. The study identified a 13% prevalence of FGM among girls and 50% among women. The prevalence of FGM among girls and women in the current study are relatively higher than the prevalence of reported in the Chad DHS [10]. The possible reason for the finding could be differences in sample size used in the current study and the Chad DHS report. For instance, while this study used a weighted sample of 6334 married and cohabiting women, the sample reported in the report in calculating FGM among girls aged 0–14 was 14,310 women of reproductive age. Among women aged 15–49, level of education, employment status, ethnicity, religion, wealth quintile and community literacy level were found as significant predictors of FGM. Age, partner’s level of education, marital status, employment status, ethnicity, religion and mother’s FGM status predicted FGM among girls aged 0–14. Aside ethnicity, the factors that predicted FGM among women and girls aged 0–14 at the same time portrayed similar patterns of influence. For instance, among women and girls aged 0–14, the odds of FGM reduced among women with higher level of education, those whose partners had higher level of education, Christians and non-working women. This is an indication that the predictors of FGM are not changing over time and hence the factors that predicted FGM among older cohorts (15–49 years) are similar to those that predict FGM among younger cohorts (0–14 years).

The results indicate that daughters of women aged 25–34 and 35 years and above were more likely to undergo FGM compared to those aged 15–24. Consistent with the findings of the current study, previous studies have also indicated that the odds of FGM among girls increases with the age of the mother [12, 18, 19]. The possible reason for this finding could be the strong connection to socio-cultural norms and practices among older women, who are more likely to uphold such norms and practices even when they have negative effects on their health [20]. Other reasons could be that older women may consider practices such as FGM as inheritance from their parents and grandparents that need to be upheld. For instance, in many high prevalent FGM societies, the practice is considered as a rite of passage to womanhood with strong ancestral and sociocultural roots [21]. Again, it is possible that the daughters of this younger group of women would not have ‘arrived’ at the age girls are commonly cut in Chad (5–14 years) as indicated in the 2013 UNICEF report on FGM [7].

The likelihood of a woman and a girl undergoing circumcision decreased among women with secondary/higher education, those whose partners had secondary/higher education and those who lived in communities with higher literacy. Similar findings on the inverse relationship between education/literacy level and FGM have been obtained in previous studies [12, 13, 18, 19, 22]. Education and literacy have been considered as essential tools for changing one’s attitude towards numerous negative socio-cultural practices, including FGM [23]. Consequently, having higher levels of education and an educated partner gives women some level of empowerment to object negative socio-cultural norms and practices including FGM of girls even when there is societal pressure to observe the practice [22]. Such empowerment is predominant in non-working women and women with rich wealth quintile, who in this study were found to be less likely to be circumcised.

Apart from women gaining empowerment though partners with higher levels of education to stop the practice of FGM, other studies have shown that FGM goes beyond geography and culture and that the practice could be influenced by patriarchal attitudes towards women and how women internalise these values. Hence, FGM and other practices are patriarchal sanctioned practices. Therefore, although FGM “appears to be a woman’s matter”, the process is influenced by patriarchy. In all of these instances, women are inflicting these harms upon their daughters or other young girls in order to ensure their future survival within their respective societies [24–26]. However, a report by UNICEF [7] has shown that the over decades view of FGM as a manifestation of patriarchal oppression of women, which would suggest that men are ardent supporters of the practice has changed and that in countries like Guinea, Sierra Leone and Chad, substantially more men than women want FGM to end. This to some extent could be as a result of high levels of education.

Ethnicity was found to be play significant role in the circumcision of women and girls in Chad. In terms of ethnicity, women who belonged to Kenemu-Borno ethnic group had the highest odds of FGM among daughters compared with those of the Sara ethnic group. The lowest risk of FGM among daughters of women belonging to Sara ethnic group could be traced to history, where the Sara ethnic group has been considered as the ethnic group with the highest level of education and highest socio-economic status, compared to the other ethnic groups in Chad [27]. Such education and socio-economic status may give women in the Sara ethnic group the empowerment to resist negative socio-cultural practices, including FGM of daughters. Surprisingly, the odds of FGM among women was higher among those of the Sara Ethnic compared to women who belonged to other ethnic groups. It is not clear why women of the Sara ethnic groups are more likely to be circumcised but less likely to have daughters who have undergone FGM. However, this can still be linked to their higher levels of education and socio-economic status which might have taken precedence over their circumcision status and influenced their decision not to circumcise their daughters.

Religion was found as a significant predictor of FGM among women and girls in Chad. With religion, Christian women were less likely to get their daughters circumcised compared to Muslim women. The findings of several studies [25, 28, 29] support the findings of the current study. This association has been deemed to be understood in line with culturally specific interpretations of religious identity [28]. Hence, despite the higher odds of FGM among Muslim women and girls aged 0–14 compared to Christians, it is possible that not all Muslim women have undergone FGM nor cut their daughters and thus the practice could be influenced by individual interpretations of religious doctrine. Although Muslim women are more likely to be circumcised and to have their daughters circumcised, the findings support the significance of a collective rather than individual Muslim identity for the discontinuation of the practice.

The results further showed women who had never experienced FGM were more likely to have their daughters circumcised. In contrast to this finding, previous studies have indicated that women who have been circumcised are more likely to condemn the practice because of its negative effects on their health [30, 31]. Further research is needed to explore the reasons why women who have been circumcised in the current study are more likely to have their daughters circumcised.

### Strengths and limitations

The strengths of the study lie in the use of a nationally representative data and large sample size that ensure reliability and generalizability of the findings to all women and girls in Chad. Notwithstanding, some limitations inherent in the current study cannot be underestimated. First, the DHS data used, followed a cross-sectional design, meaning that causal inferences drawn from the findings should be interpreted with caution. At best, the findings should be considered as explaining associations, meaning that casual inferences cannot be drawn. Secondly, collecting data on a sensitive issue like FGM using self-report could be influenced by social desirability bias. Thirdly, most of the girls aged 0–14 may not arrive at cutting age at the time of the survey and this can also lead to under-reporting the prevalence of FGM among girls. Finally, using secondary data, which contains already existing variables, makes it less feasible to explore interesting socio-cultural factors that may influence FGM among women and girls.

## Conclusion

This study has identified several individual and contextual factors as predictors of FGM among women and girls in Chad. The findings imply the need to adopt strategies aimed at addressing these factors in order to help eliminate the practice of FGM. Government and non-governmental organisations in Chad need to implement policies that enhance media advocacy and community dialogue to help deal with FGM in the country. Future studies should examine the attitudes of women of Chad towards FGM and the factors that account for the attitudes. Also, there is the need for studies that employ qualitative research methods to compliment this type of analysis and help to explain why women who have been circumcised in the current study are more likely to have their daughters circumcised.

## Data Availability

The data can be accessed at https://dhsprogram.com/what-we-do/survey/survey-display-465.cfm.

## Abbreviations

AIC: Akaike’s Information Criterion
aOR: adjusted odds ratio
CIs: Confidence intervals
DHS: Demographic and Health Survey
FGM: Female genital mutilation/cutting
LLR: Log-likelihood ratio
UNICEF: United Nations Population Fund
VIF: Variance inflation factor
